# Endothelial cell Piezo1 mediates pressure-induced lung vascular hyperpermeability via disruption of adherens junctions

**DOI:** 10.1073/pnas.1902165116

**Published:** 2019-06-11

**Authors:** Emily E. Friedrich, Zhigang Hong, Shiqin Xiong, Ming Zhong, Anke Di, Jalees Rehman, Yulia A. Komarova, Asrar B. Malik

**Affiliations:** ^a^Department of Pharmacology, University of Illinois College of Medicine, University of Illinois at Chicago, Chicago, IL 60612;; ^b^Center for Lung and Vascular Biology, University of Illinois College of Medicine, University of Illinois at Chicago, Chicago, IL 60612

**Keywords:** endothelial, permeability, Piezo1

## Abstract

Increased pulmonary microvessel pressure experienced in left heart failure, head trauma, or high altitude can lead to endothelial barrier disruption referred to as capillary “stress failure” that causes leakage of protein-rich plasma and pulmonary edema. However, little is known about vascular endothelial sensing and transduction of mechanical stimuli inducing endothelial barrier disruption. Piezo1, a mechanosensing ion channel expressed in endothelial cells (ECs), is activated by elevated pressure and other mechanical stimuli. Here, we demonstrate the involvement of Piezo1 in sensing increased lung microvessel pressure and mediating endothelial barrier disruption. Studies were made in mice in which Piezo1 was deleted conditionally in ECs (*Piezo1*^*iΔEC*^), and lung microvessel pressure was increased either by raising left atrial pressure or by aortic constriction. We observed that lung endothelial barrier leakiness and edema induced by raising pulmonary microvessel pressure were abrogated in *Piezo1*^*iΔEC*^ mice. Piezo1 signaled lung vascular hyperpermeability by promoting the internalization and degradation of the endothelial adherens junction (AJ) protein VE-cadherin. Breakdown of AJs was the result of activation of the calcium-dependent protease calpain and degradation of the AJ proteins VE-cadherin, β-catenin, and p120-catenin. Deletion of Piezo1 in ECs or inhibition of calpain similarly prevented reduction in the AJ proteins. Thus, Piezo1 activation in ECs induced by elevated lung microvessel pressure mediates capillary stress failure and edema formation secondary to calpain-induced disruption of VE-cadherin adhesion. Inhibiting Piezo1 signaling may be a useful strategy to limit lung capillary stress failure injury in response to elevated vascular pressures.

## Significance

Increased hydrostatic pressure in lung capillaries experienced during high altitude, head trauma, and left heart failure can lead to disruption of lung endothelial barrier and edema formation. We identified Piezo1 as a mechanical sensor responsible for endothelial barrier breakdown (barotrauma) secondary to reduced expression of the endothelial adherens junction proteins VE-cadherin, β-catenin, and p120-catenin. Endothelial-specific deletion or pharmacological inhibition of Piezo1 prevented lung capillary leakage, suggesting a therapeutic approach for preventing edema and associated lung failure.

Lung fluid homeostasis is dependent on the integrity of the lung endothelial barrier (1). Barrier breakdown results in protein-rich pulmonary edema formation through increased flux of fluid and plasma proteins across pulmonary capillaries (2). High-permeability pulmonary edema is due to disruption of endothelial adherens junctions (AJs), which consist of the transmembrane adhesive protein VE-cadherin associated with β-catenin, α-catenin, and p120-catenin (3, 4). Lung endothelial permeability, although stringently regulated, can increase in response to pathogens, humoral mediators, activation of inflammatory cells, and unusually high capillary hydrostatic pressure (5). West and colleagues (6–9) have described the phenomenon of “stress failure” of pulmonary capillaries to explain severe endothelial capillary breakdown in response to high pulmonary vascular pressures that can lead to high-altitude pulmonary-, neurogenic-, and left heart failure-associated pulmonary edema (10–13). It is known that disruption of the thin capillary–alveolar barrier (<1 µm) comprising alveolar epithelial cell and endothelial cell (EC) monolayers can be induced by high lung capillary pressures (14, 15); however, it is not known whether endothelial barrier breakdown induced by the pressure rise is the result of activation of intrinsic endothelial signaling pathways that cause disassembly of AJs or release of permeability-increasing mediators.

Piezo1, a 286-kDa transmembrane cation channel (16–19), is gated by membrane tension and changes in membrane curvature such as induced by high pressure, which activate the influx of cations and downstream signaling pathways (20, 21). Endothelial-specific disruption of *Piezo1* in mice was shown to impair vascular development in response to shear stress secondary to defective alignment of ECs (16). Deletion of Piezo1 also prevented shear stress-induced sprouting angiogenesis (22). In addition, endothelial-expressed Piezo1 can sense disturbed blood flow and is linked to inflammatory signaling and atherosclerosis progression (23). These studies show an important role of Piezo1 in regulating EC function and vascular homeostasis.

Here, we addressed the possibility that Piezo1 sensing of high vascular pressures at the lung endothelial surface mediates disassembly of AJs. AJs are the primary paracellular route for the exchange of fluid and protein across the vessel wall, and their disruption increases endothelial permeability through reduction of VE-cadherin homotypic interactions (24). We demonstrated that Piezo1 is the mechanical sensor responsible for hydrostatic pressure-induced endothelial barrier breakdown that occurred secondary to reduced VE-cadherin homotypic interaction leading to disruption of AJs.

## Results

### Piezo1 Mediates Increased Lung Endothelial Permeability in Response to Vascular Pressure Rise.

**Fig. 1.**
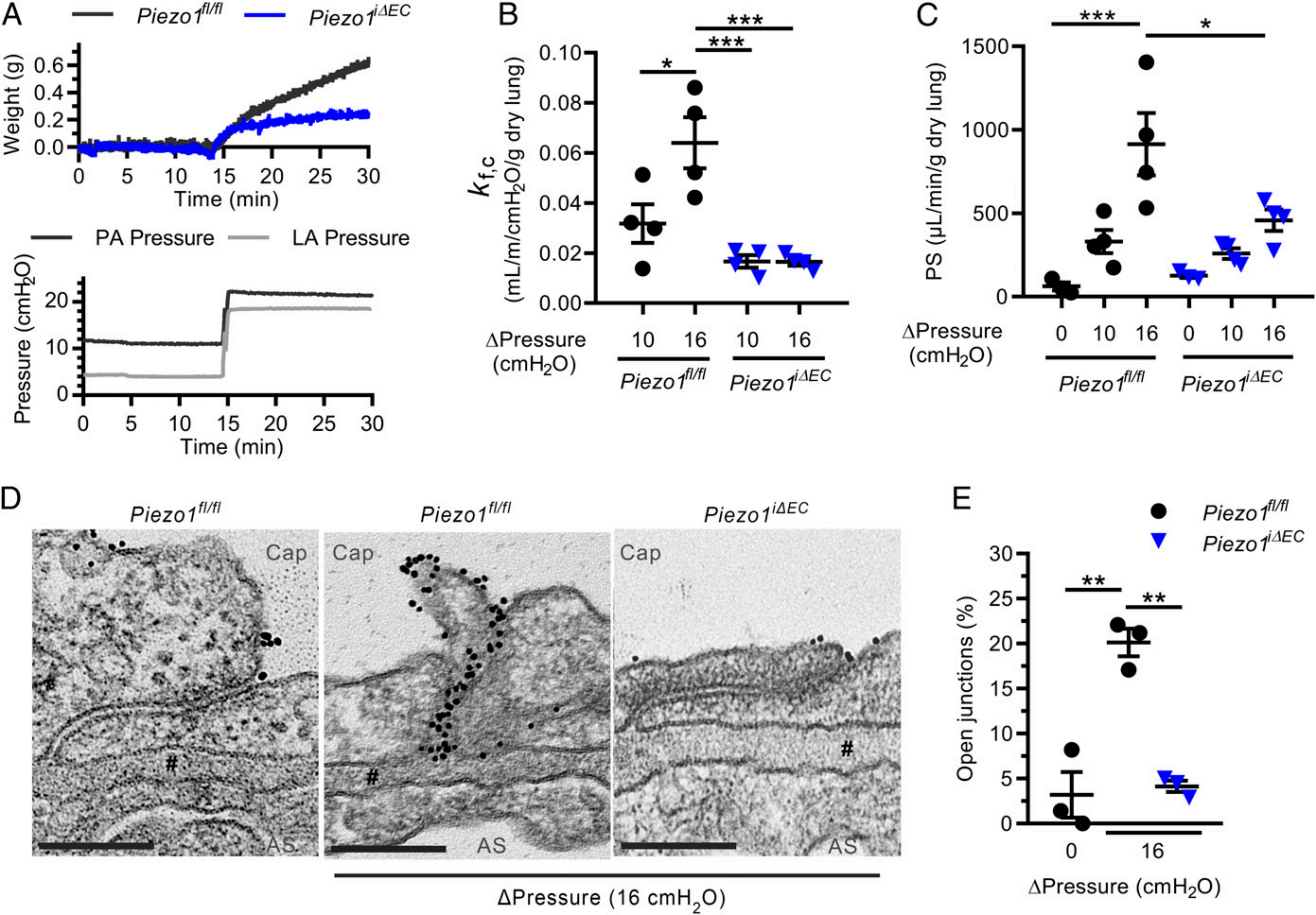
Elevated pulmonary microvessel pressure increases lung vascular permeability by a Piezo1-dependent mechanism. (*A*) Changes in lung wet weight and pulmonary artery pressure in response to increased left atrial pressure in lungs from *Piezo1*^*fl/fl*^ and *Piezo1*^*iΔEC*^ mice. (*B* and *C*) Increases in capillary filtration coefficient (*K*_f,c_) (*B*) and pulmonary transvascular permeability (PS) to albumin tracer (*C*) in response to increased left atrial pressure in lungs from *Piezo1*^*fl/fl*^ and *Piezo1*^*iΔEC*^ mice. (*D*) Transmission electron microscopy of interendothelial junctions in lung capillaries of *Piezo1*^*fl/fl*^ and *Piezo1*^*iΔEC*^ mice subjected to increased left atrial pressure; representative images from *n* = 3 mice. Lung vessels were subjected to the same rise in left atrial pressure as in *A*. Cap, capillary lumen; AS, alveolar space; **#**, basement membrane. (Scale bar, 0.2 µm.) (*E*) Percentage of junctions with gold–albumin particles for each condition. Data are shown as mean ± SEM; *P ≤ 0.05; **P ≤ 0.01; ***P ≤ 0.001.

To address the role of EC-expressed Piezo1 in mediating endothelial barrier failure, we generated a genetic mouse model of inducible deletion of *Piezo1* gene in ECs (*Piezo1*^*iΔEC*^) using the endothelial-specific Cre/loxP recombination system (25, 26). Deletion of Piezo1 in ECs by treating mice with tamoxifen was validated by qPCR and Western blot analysis of lung EC lysates (*SI Appendix*, Fig. S1). The relationship between pulmonary capillary pressure and endothelial permeability to both fluid and albumin was determined in mouse lungs (27). We observed that the rise in lung capillary pressure induced by a rise in left atrial pressure increased lung wet weight in *Piezo1*^*fl/fl*^ control mice, whereas EC deletion of Piezo1 (*Piezo1*^*iΔEC*^ lungs) markedly reduced the increase in lung wet weight (Fig. 1*A*). Lung capillary filtration coefficient (*K*_f,c_), a measure of vessel wall permeability to fluid, increased significantly in *Piezo1*^*fl/fl*^ mice, whereas the response was inhibited in *Piezo1*^*iΔEC*^ mice (Fig. 1*B*). Pulmonary transvascular albumin permeability, measured as the permeability surface area product (PS) (27), was significantly greater in *Piezo1*^*fl/fl*^ mice than in *Piezo1*^*iΔEC*^ mice at all levels of hydrostatic pressure (Fig. 1*C*), indicating the essential role of Piezo1 in mediating pressure-induced increase in pulmonary transvascular albumin flux.

To identify the route of albumin leak at the level of AJs, we carried out transmission electron microscopy studies of lung endothelium (28). We observed an 8-fold increase in the number of open AJs, defined by the presence of 6- to 9-nm colloidal gold–albumin tracers, in AJs of pulmonary capillaries of *Piezo1*^*fl/fl*^ control mice subjected to the rise of capillary pressure (Fig. 1 *D* and *E*). In contrast, AJs showed the characteristic restrictive barrier in *Piezo1*^*iΔEC*^ mice (Fig. 1 *D* and *E*). These findings demonstrate that pressure-induced Piezo1 was responsible for opening of the paracellular permeability route in control mice.

### Piezo1 Activation Induces Internalization of VE-Cadherin.

**Fig. 2.**
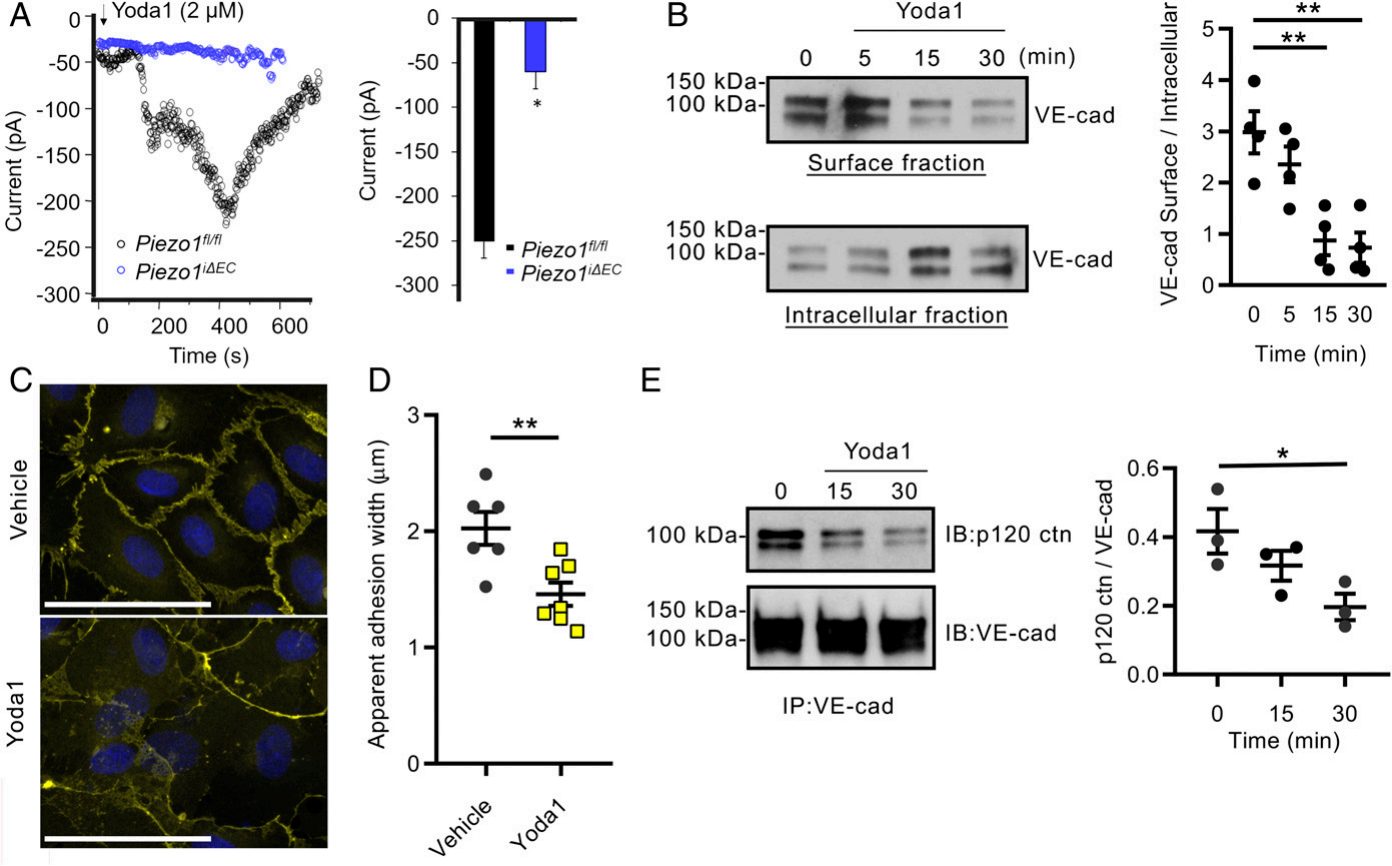
Activation of Piezo1 disrupts VE-cadherin junctions in EC monolayers. (*A*) Time course of whole-cell inward current development (*Left*) and summary of the peak inward current (*Right*) induced by extracellular application of Yoda1 in lung ECs from both *Piezo1*^*fl/fl*^ and *Piezo1*^*iΔEC*^ mice. Inward currents were elicited by −110 mV voltage. (*B*) Activation of Piezo1 with 5 µM Yoda1 induces VE-cadherin internalization in human lung microvascular ECs. Western blot analysis of biotinylated VE-cadherin at plasma membrane and in cytosol (*Left*), and quantified as a ratio of surface-to-intracellular fractions (*Right*). (*C*) Decreased accumulation of VE-cadherin (yellow) at AJs in EC monolayers treated with Yoda1 for 30 min. (Scale bar, 100 µm.) (*D*) Quantification of data in *C*. (*E*) Yoda1 activation of Piezo1 decreased p120-catenin association with VE-cadherin as shown by coimmunoprecipitation assay (*Left*) and quantified as a ratio of p120-catenin to VE-cadherin (*Right*). Individual data points shown with mean ± SEM; *P ≤ 0.05; **P ≤ 0.01.

To address mechanisms of Piezo1-mediated increase in lung vascular permeability, we studied changes in VE-cadherin expression in confluent lung EC monolayers. Here, we used Yoda1, a specific activator of Piezo1 (29), to assess mechanisms of Piezo1-induced disruption of AJs. First, the specificity of Yoda1 in mediating Piezo1 activation was determined using patch-clamping analysis of ECs isolated from *Piezo1*^*fl/fl*^ and *Piezo1*^*iΔEC*^ mouse lungs. We observed that Yoda1 activated cation influx in *Piezo1*^*fl/fl*^ control ECs, whereas the response was blocked in *Piezo1*^*iΔEC*^ mouse ECs (Fig. 2*A*). Treating human EC monolayers with Yoda1 significantly reduced plasma membrane-associated VE-cadherin in a time-dependent manner as assessed by the cell surface biotinylation assay, while increasing the cytosolic pool of VE-cadherin (Fig. 2*B*). Activation of Piezo1 in endothelial monolayers also reduced VE-cadherin expression at AJs (Fig. 2 *C* and *D*), indicating that Piezo1 induced the internalization of VE-cadherin from AJs. Consistent with this observation, the activation of Piezo1 with Yoda1 markedly reduced the association of VE-cadherin with p120-catenin, the key protein of the AJ complex regulating internalization of VE-cadherin (30) (Fig. 2*E*). In response to Yoda1, we also detected an increase in a 90-kDa VE-cadherin degradation product (*SI Appendix*, Fig. S2), similar to the proteolytic processing of VE-cadherin occurring during its endocytosis (31). These results are in agreement with the reported effects of Yoda1 in decreasing VE-cadherin staining in lymphatic EC junctions (32) and the hydrostatic pressure-induced reduction in VE-cadherin expression in ECs (33, 34).

We next addressed whether genetically stabilizing AJs by preventing VE-cadherin endocytosis reduces the high vascular pressure-induced disruption of VE-cadherin junctions shown in Fig. 1 *D* and *E*. Here, we used the knockin mouse model expressing the genetically engineered fusion proteins VE-cadherin–F506 and vascular endothelial protein tyrosine phosphatase (VE-PTP)–FRB* under the control of VE-cadherin promoter (35). Treatment of these mice with rapamycin stabilizes the interaction of VE-cadherin and VE-PTP through the binding of FK506 and FRB* (*SI Appendix*, Fig. S3) and prevents VE-cadherin internalization (35). We observed that stabilizing VE-cadherin and VE-PTP interaction at AJs through preventing VE-cadherin endocytosis in these mice significantly reduced pressure-induced increase in lung vascular permeability to both albumin and fluid (*SI Appendix*, Fig. S3); thus, these findings indicate the importance of VE-cadherin internalization as a central mechanism mediating lung endothelial hyperpermeability induced by the rise in hydrostatic pressure.

### Increased Lung Capillary Pressure Induces Piezo1-Dependent Vascular Hyperpermeability In Vivo.

**Fig. 3.**
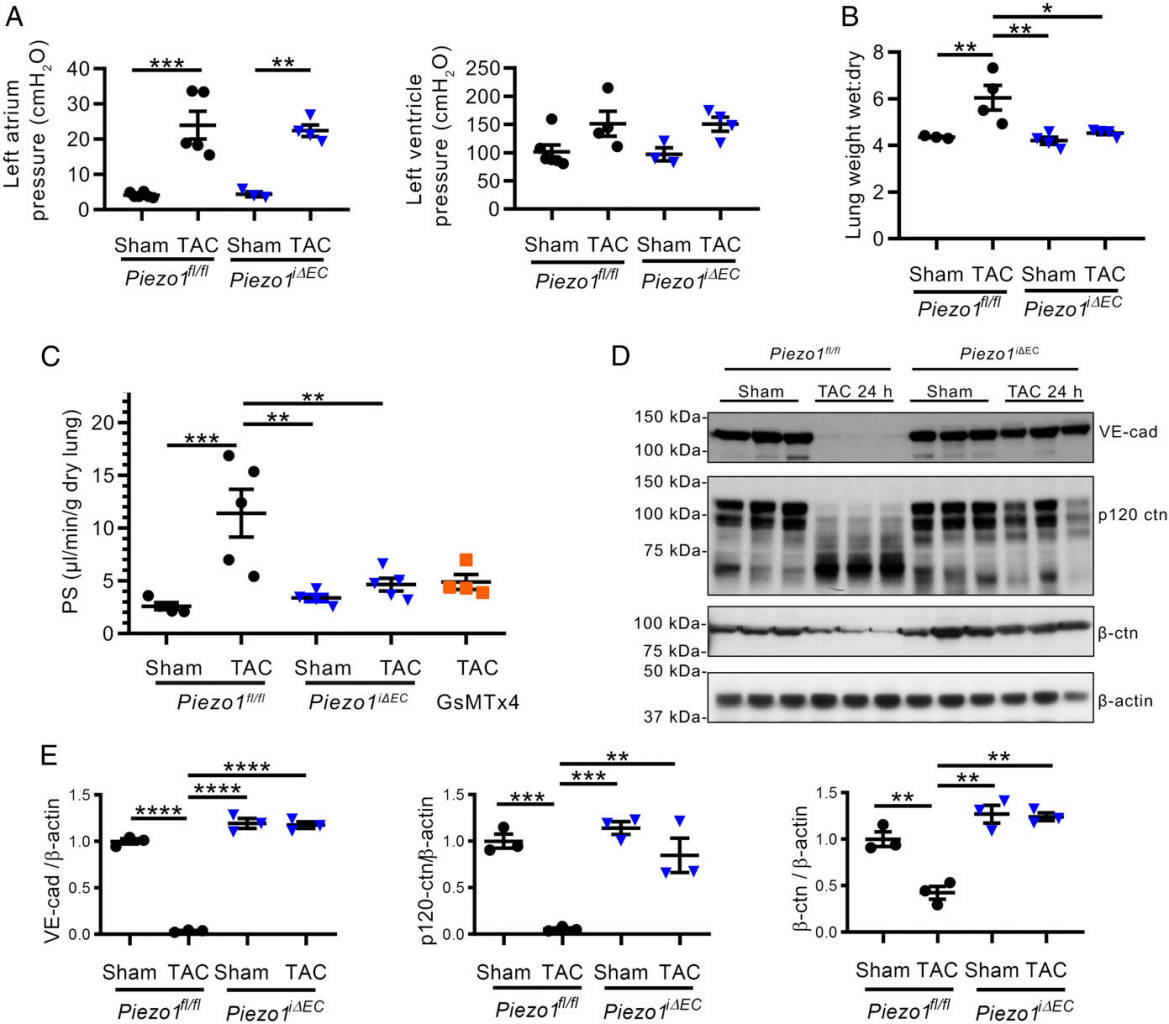
Increased lung microvessel pressure induces Piezo1-dependent lung vascular hyperpermeability secondary to reduced expression of AJ proteins. (*A*) Effects of 50% constriction of aorta (TAC) in *Piezo1*^*fl/fl*^ and *Piezo1*^*iΔEC*^ mice on left atrial pressure (*Left*) and left ventricular systolic pressure (*Right*) recorded at 24 h post-TAC. (*B* and *C*) Accumulation of lung water (*B*) and increased lung transvascular albumin permeability (PS) (*C*) were blocked in *Piezo1*^*iΔEC*^ mice and in *Piezo1*^*fl/fl*^ mice treated with GsMTx4 at 24 h post-TAC. (*D* and *E*) Loss of VE-cadherin, β-catenin, and p120-catenin at 24 h post-TAC. Individual data points are shown as well as mean ± SEM; *P ≤ 0.05; **P ≤ 0.01; ***P ≤ 0.001; ****P ≤ 0.0001.

We next investigated the functional relevance of the mechanosensor Piezo1 in mediating increased lung vascular permeability using a mouse model of sustained increase in pulmonary vascular pressures. Here, studies were made in the transverse aortic constriction (TAC) mouse model (36) in which the aorta is constricted ∼50% through surgery. This resulted in left ventricular systolic and left atrial pressure increases 24 h after surgery that were similar in *Piezo1*^*fl/fl*^ and *Piezo1*^*iΔEC*^ mice (Fig. 3*A*). First, we noted that *Piezo1*^*iΔEC*^ mice subjected to TAC showed increased survival compared with *Piezo1*^*fl/fl*^ mice subjected to TAC (*SI Appendix*, Fig. S4). Increased pulmonary vascular pressures in *Piezo1*^*fl/fl*^ mice were associated with significant pulmonary edema (Fig. 3*B*) and increased vascular permeability (Fig. 3*C*); however, endothelial-specific deletion of Piezo1 prevented both responses (Fig. 3 *B* and *C*).

To assess whether pharmacologically inhibiting Piezo1 phenocopied the response seen in *Piezo1*^*iΔEC*^ mice, we used mechanotoxin-4 (also known as GsMTx4), an inhibitor of Piezo1 (37), and observed that GsMTx4 reduced pulmonary transvascular albumin permeability in *Piezo1*^*fl/fl*^ TAC mice to the same level as in *Piezo1*^*iΔEC*^ mice (Fig. 3*C*). Furthermore, the increase in lung vascular pressure post-TAC reduced the expression of VE-cadherin, β-catenin, and p120-catenin proteins in lungs (*SI Appendix*, Fig. S5), which remained low during the 24-h post-TAC period (Fig. 3 *D–F*). A 70-kDa cleavage product of p120-catenin was observed post-TAC, consistent with calpain-mediated cleavage of p120-catenin (38). Reduced VE-cadherin, p120-catenin, and β-catenin protein expression was prevented in *Piezo1*^*iΔEC*^ mice and by GsMTx4-mediated inhibition of Piezo1 (*SI Appendix*, Fig. S5), indicating the requisite role of Piezo1 in signaling the pressure-induced disruption of VE-cadherin junctions.

### Piezo1-Induced Calpain Activation Disrupts VE-Cadherin Homotypic Interaction.

**Fig. 4.**
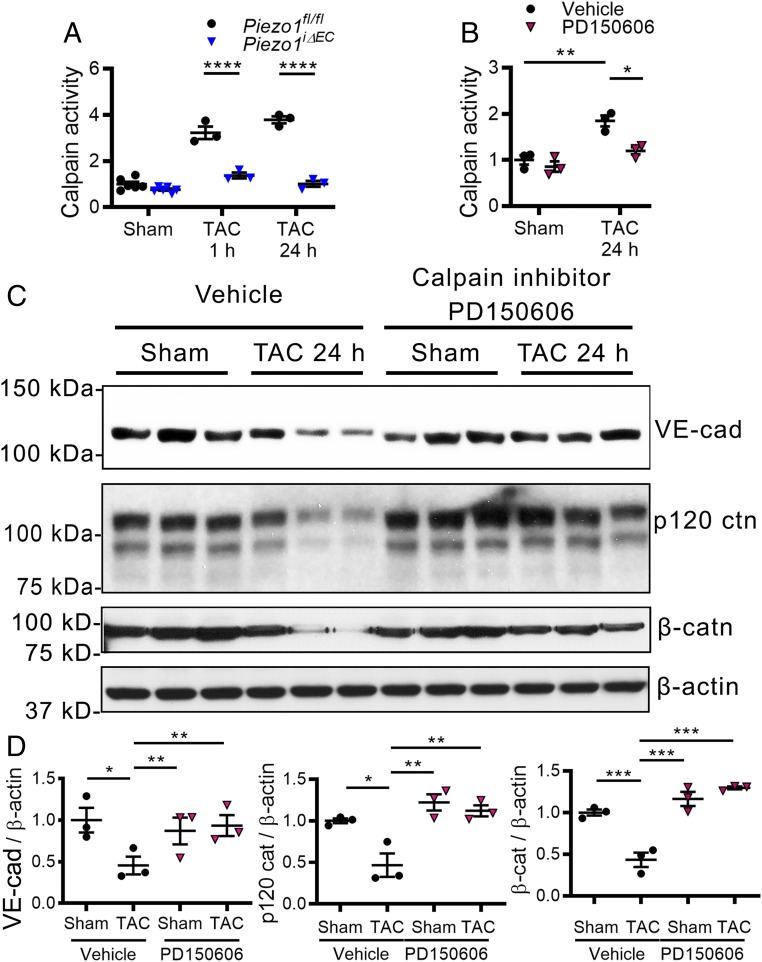
Piezo1-dependent activation of calpain cleaves junctional proteins in lung ECs of mice in response to elevated lung microvessel pressure. (*A*) Increased activity of calpain was blocked in *Piezo1*^*iΔEC*^ mice at 1 and 24 h post-TAC. (*B*–*D*) Treatment of mice with calpain inhibitor PD150606 reduced calpain activity after TAC (*B*) and prevented the loss of VE-cadherin, β-catenin, and p120-catenin in lung vessels (*C* and *D*). Individual data points are shown as well as mean ± SEM; *P ≤ 0.05; **P ≤ 0.01; ***P ≤ 0.001; ****P ≤ 0.0001.

Because Piezo1 can activate calpain (16), we next investigated the possible role of calpain in cleaving the AJ proteins in response to the pressure rise. We observed significantly increased calpain activity in *Piezo1*^*fl/fl*^ mice post-TAC, whereas this response was blocked in *Piezo1*^*iΔEC*^ mice (Fig. 4*A*). We also treated *Piezo1*^*fl/fl*^ mice with the calpain inhibitor PD150606 (39) and observed decreased calpain activity post-TAC as compared with vehicle-treated mice (Fig. 4*B*). In addition, we observed that inhibition of calpain prevented the degradation of AJ proteins post-TAC (Fig. 4 *C* and *D*), suggesting that Piezo1 activation of calpain and the proteolysis of these proteins were responsible for disruption of AJs.

## Discussion

Here, we showed the essential role of Piezo1 sensing of elevated hydrostatic pressure in lung microvessel endothelia in increasing endothelial permeability via the paracellular AJ route. VE-cadherin, the primary adhesion protein at AJs regulating endothelial permeability, was a key downstream target of pressure-induced activation of Piezo1. Our results using EC-specific Piezo1-deleted mice thus demonstrate that lung vascular hyperpermeability following increased capillary pressure, or stress failure of thin-walled pulmonary capillaries described by West et al. (6, 7, 12, 14, 40, 41), is the result of Piezo1 activation and the breakdown of lung endothelial AJs and opening of the paracellular route.

Our results showed that the endothelial barrier was disrupted through Piezo1-dependent activation of the protease calpain and subsequent proteolysis of the junctional proteins VE-cadherin, β-catenin, and p120-catenin. As calpain activation requires calcium signaling (42), calpain activation may involve Piezo1-mediated calcium influx in ECs (16, 32). The proteolytic calpain isoforms µ-calpain and m-calpain are both expressed in ECs, and thus both may be involved in proteolytic cleavage of junctional proteins (31). It has been reported that m-calpain cleaves VE-cadherin between the juxtamembrane domain and the catenin-binding domain (CBD), resulting in a 90-kDa fragment (43). We observed the appearance of a similar-sized fragment in response to Piezo1 activation. Also, µ-calpain cleaves VE-cadherin to a 90-kDa VE-cadherin product (31), suggesting that both calpain isoforms may be involved.

We showed that genetic stabilization of VE-cadherin at the junctions through inhibition of its endocytosis machinery (35) prevented the pressure-induced increase in lung vascular hyperpermeability. The uncoupling of p120-catenin from VE-cadherin engages the VE-cadherin endocytosis machinery and signals the internalization of VE-cadherin (30). p120-catenin thus establishes the set point for VE-cadherin cell surface expression because of its central role in stabilizing VE-cadherin at the junctions (44); therefore, proteolysis of p120-catenin may be another factor in promoting internalization and loss of VE-cadherin at AJs (44). Thus, Piezo1-induced VE-cadherin endocytosis from AJs appeared to be an important step before calpain-mediated proteolysis of VE-cadherin within the CBD. VE-cadherin, subsequent to its degradation, is then transported to lysosomes (31, 45).

Although the focus of our studies was on Piezo1 expressed in ECs, our results do not preclude the involvement of other mechanosensitive channels or compensating ion fluxes as factors responsible for pressure-induced endothelial hyperpermeability. Transient receptor potential cation channel subfamily V member 4 (TRPV4), another mechanosensitive cation channel (46), may also be involved. Blocking TRPV4 reduced pulmonary edema in response to increased pulmonary venous pressure (46), although it was not evident whether this was secondary to stabilization of VE-cadherin junctions, as was the case with deletion or blockade of Piezo1.

The finding that pressure-induced activation of Piezo1 disrupted the endothelial junctional barrier raises the possibility of targeting Piezo1 to prevent leaky lung microvessels and edema formation in conditions such as cardiogenic pulmonary edema, which is due to left heart failure, or hydrostatic pressure-induced breakdown of lung endothelial barrier associated with high altitude-induced pulmonary edema or head trauma (the Cushing response) (47). The blood–gas barrier in alveolar capillaries of mammals is only ∼0.3 µm thick (48). Stress failure at these sites was seen in several species but it was reversed in time (14, 49). As to the physiological significance of pressure-induced disruption of endothelial junctions, we surmise that hyperpermeability of lung capillaries induced by Piezo1 represents an “escape valve” that may prevent catastrophic damage to the endothelium due to pulmonary barotrauma.

## Materials and Methods

Mice were bred and maintained under specific pathogen-free conditions at the University of Illinois at Chicago animal facility, and all protocols were approved by the Animal Care Committee administered through the Office of Animal Care and Institutional Biosafety.

Complete methods can be found in *SI Appendix*, *Materials and Methods*.

## Supplementary Material

Supplementary File
