# Supplementary material for: Endothelial cell Piezo1 mediates pressure-induced lung vascular hyperpermeability via disruption of adherens junctions
Source: Proc Natl Acad Sci U S A. 2019 Jun 11;116(26):12980–5. doi: 10.1073/pnas.1902165116 (PMC6600969; doi:10.1073/pnas.1902165116)
Supplement: Supplementary File [file pnas.1902165116.sapp.pdf]

PNAS
www.pnas.org

Supplementary Information for

Endothelial Cell Piezo1 Mediates Pressure-Induced Lung Vascular Hyper-Permeability via Disruption of Adherens Junctions

Emily E. Friedrich[†], Zhigang Hong[†], Shiqin Xiong[†], Ming Zhong, Anke Di, Jalees Rehman, Yulia A. Komarova, Asrar B. Malik[*]

Paste corresponding author name here Asrar B. Malik
Email: abmalik@uic.edu
and Yulia A Komarova
Email: ykomarov@uic.edu

This PDF file includes:

Supplementary text
Figs. S1 to S5
References for SI reference citations

www.pnas.org/cgi/doi/10.1073/pnas.1902165116

Supplementary Information Text

Materials and Methods

Mouse strains and models of increased pulmonary capillary pressure Mice were bred and maintained under specific pathogen–free conditions at the University of Illinois at Chicago animal facility and approved by the Animal Care Committee. Endothelial cell-specific Piezo1–knockout mice were generated by backcrossing Piezo1$^{flox/flox}$ mice with Endo-SCL-Cre mice, which express an endothelial-specific Cre in the adult.(1) Piezo1flox/flox mice was a kind gift from Dr. David J. Beech of the University of Leeds, Leeds, UK.(2)

Genotyping primer sequences for SCL-Cre (482bp):
F- TCGATGCAACGAGTGATGAG; R-TTCGGCTATACGTAACAGGG.
Primer for Cre deletion-spanning analysis (379bp):
F- ACCACCTGAGAAGTTGTCCC; R-ACTCATCTGGGTGAGGTTGC.
Primer for LoxP-spanning (155, 189bp):
F-GGAGGGTTGCTTGTTGGATA, R-ACTCATCTGGGTGAGGTTGC.

Floxed littermates (Piezo1$^{fl/fl}$) that were null for Cre gene were used as controls. Piezo1 deletion in adult 4 wk old mice were induced by tamoxifen administration (intraperitoneal 2 mg/day for 5 days).

Knock-in mice expressing the fusion proteins VE-cadherin-FK 506 binding protein and VE-PTP-FRB* under the endogenous VE-cadherin promoter were a gift from Dr. Dietmar Vestweber. Rapamycin, a small molecule inhibitor of mTor, binds FK 506 binding protein and FRB* domains, stabilizing the interaction of VE-cadherin and vascular endothelial protein tyrosine phosphatase (VE-PTP) to prevent VE-cadherin dissociation from VE-PTP. (3) Homozygous Knock-in mice were injected intravenously either with control vehicle or with rapamycin 14 and 2 h before the experiment. Both male or female mice (8 to 12 weeks old) were used for all experiments.

Measurement of pulmonary vessel filtration coefficient Pulmonary vessel filtrations coefficients were measured as previously described by us.(4) Mice were anesthetized with an intraperitoneal injection of ketamine (100 mg/kg body weight) and xylazine (15 mg/kg body weight). A midline incision was made along the ventral surface of the neck to expose the trachea. The trachea was cannulated for artificial ventilation (120 breaths/min; peak inspiratory pressure, 10 cm H_2O; and end expiratory pressure, +2 cm H_2O) with the anesthetic gas mixture. Then, heparin sodium (700 U/kg) was administered via the right jugular vein. Next, a thoracotomy was carried out. The

pulmonary artery was cannulated rapidly; a second cannula was inserted into the left atrium for drainage of the pulmonary venous effluent. Lung and heart were removed en bloc from the thorax and suspended from a counter-weighted beam balance. Lung was perfused at constant flow (2 mL/min), temperature (37°C) and venous pressure (4 cmH_2O). Microvascular filtration coefficient ($K_{f,c}$) was measured from the rate of lung wet weight gain after a step increase (10 or 16 cmH_2O) in venous pressure for 20 minutes. The rate of weight gain was normalized by the lung dry weight and step size to calculate $K_{f,c}$ in units of milliliters per minute per centimeters of water per gram.(4)

Ex Vivo Albumin PS product measurement. Mouse lungs were perfused as described above without recirculation to measure albumin permeability × surface area (PS product), as previously described by us.(5) After a 20-min equilibration perfusion, measurements were made after a step increase (0, 10 or 16 cmH_2O) in venous pressure for 5 minutes. Then, ^{125}I-labeled albumin (final activity of 60,000 cpm/mL, Anazao Health Corp, Tampa, FL) was infused into the perfusing liquid for 3 min via a side-arm in the pulmonary artery cannula. A sample of the venous effluent was collected. Vascular tracer was washed out for 6 min. The venous effluent sample and the entirety of lung tissue were counted for gamma-radioactivity. Albumin PS was computed in units of mL/min/g from A/ct, where A represents the lung counts (cpm/g dry lung), c, the venous effluent tracer concentration (cpm/mL), and t, the exposure time (min).

In Vivo Albumin PS product measurement Mice were anesthetized with an intraperitoneal injection of ketamine (100 mg/kg body weight) and xylazine (15 mg/kg body weight) after 24 hr sham or TAC surgery. 100 ul 125I-labeled albumin (10 uCi/mL) was injected into the left jugular vein. After 30 min post-injection, lungs were briefly flushed to eliminate vascular tracer and was counted for gamma-radioactivity. Blood sample was collected and radioactivity was counted for comparison. Albumin PS was computed in units of mL/min/g from A/ct, where A represents the lung counts (cpm/g dry lung), c, Blood concentration (cpm/mL), and t, the exposure time (min). (5)

Transverse Aortic Constriction (TAC) Minimally invasive transverse aortic constriction was performed under 1.5% isoflurane mixed with 0.5 -1.0 L/min room air.(6) Under microscopic view, a 2 cm midline incision till the second rib was performed in the anterior neck. The sternum was subsequently cut down to the second rib level. The aortic arch was reached simply by separating the connective tissues and thymus. A 6-0 silk suture underneath the aortic arch between the innominate and left carotid arteries was tied against 25 G needle with a loose double knot. Then immediately the needle will be removed, the lungs re-inflated and the chest closed

layer-by-layer with 6-0 silk suture. Implementation of outlined surgical procedure in its entirety was taken no more than 30 min. The success of TAC was confirmed by echocardiography, which determine the flow velocities ratio change from the left to right carotid arteries, the dimension of the blood vessels, and the left ventricular function. In the GsMTx4 group, 3 μM GsMTx4 (Tocris Bioscience) was IV administrated 30 minutes before the TAC surgery. Dosing of GsMTx4 based on previous studies with the peptide.(7) For calpain inhibition studies, 8 μmole/kg PD150606 was administered IP 30 min before TAC surgery.

In vivo measurement of left atrial pressure and left ventricular pressure The left atrial pressure and left ventricular pressure was measured 24 h after sham or TAC surgery. Mice were anesthetized with an intraperitoneal injection of ketamine (100 mg/kg body weight) and xylazine (15 mg/kg body weight), and quickly intubated and ventilated with a respirator. A horizontal incision was made inferior to the sternum and xiphoid process. The rib cage was slightly retracted, and the diaphragm was gently cut away to attain an apical view of the myocardium. A 27-gauge needle was inserted into the apex of the LV free wall to create a guide-hole. A 1.4F The SPR-671 Mikro-Tip® mouse Millar pressure catheter (Houston, Texas) was then inserted into the lumen of LV, and further though the mitral valve (when it was open) to left atrium, to assess in real-time left atrial and left ventricular pressure. The data were recorded through ADInstruments PowerLab Data Acquisition Systems (Colorado Springs, CO).

Western blot analysis Lung samples were mechanically homogenized in lysis buffer containing protease inhibitors. Blots were probed with anti-VE-cadherin (ab33168, abcam) and anti-β-actin (13E5, Cell Signaling).

Transmission Electron Microscopy Approximately 6-9 nm colloidal gold-albumin complexes were prepared as tracers to determine the route of protein leak across the blood gas barrier as previously described (8). Approximately 6-9 nm colloidal gold was prepared by heating separately 80 mL 0.35 mM $HAuCl_4$ and 20 mL 0.2% trisodium citrate, 0.1% tannic acid and 2.5 mM K_2CO_3 to 60 C before mixing the two solutions together with stirring and then heating to a rapid boil for 20 min. The solution cooled for 1 h at 4 C before stabilizing with 125 μg/mL linoleic acid-oleic acid albumin (L9655, Sigma) and 200 μg/mL polyglutamic acid. Solutions were ultracentrifuged at 105,000 x g for 1.5 h at 4 C. Supernatant was discarded. Colloidal gold-albumin was then diluted to $A_{520} = 1$ in Hanks' Balanced Salt Solution (HBSS) with calcium and magnesium just before use.

Mice were anesthetized, and the pulmonary artery and left atrium were cannulated as described above, but without removed the heart and lungs. Flow rate was held between 2.0 to 2.2

mL/min for all animals. The vasculature was first precleared with HBSS with calcium and magnesium at. The pressure was elevated by 16 cmH_2O for 15 min and the perfusate was simultaneously changed to the gold-albumin containing buffer. This was followed by a 3 min wash with HBSS. Mouse lungs were then fixed by *ex vivo* perfusion with 2.5% glutaraldehyde, 4% PFA, 0.1 M HEPES and 2 mM $CaCl_2$. The pressure for washing and fixation steps remained elevated for experimental groups or kept at baseline for control groups.

Immediately following fixation, lungs were dissected and minced to 1 mm pieces and placed back in fixative overnight at 4 C. Samples were washed in 0.1 M sodium cacodylate buffer for 30 min twice at room temperature and post-fixed with Palade's buffer, consisting of 0.04 M acetate buffer (#11482-56, EMS), 1% OsO_4 (#19150, EMS), 0.02 N HCl for 1 h on ice and protected from light. Samples were then submerged in Kellenberger's buffer, which consisted of 0.04M acetate buffer, 0.028 N HCl and 5 mg/mL uranyl magnesium acetate (#22500, EMS) overnight at 4 C and protected from light. Samples were then dehydrated through a graded series of ethanol for 5 min each in 50%, 70% and 95% ethanol and for 10 min, twice in 100 % ethanol. Samples were further dehydrated in propylene oxide twice for 15 min, before storing in a 1:1 mixture of Embed-812/DER736 (14130, EMS) embedding reagent and propylene oxide overnight. Samples were then placed in embedding reagent for at 6 h before embedding in molds. Samples in embedding reagent in molds were cured at 60 C for 3 days. Resultant blocks were thin sectioned, and grids were post-stained. Grids were Imaged using a JOEL JEM 1220 TEM fitted with a Gatan digital camera. Grids from three blocks were randomly chosen from each of n = 3 mice per treatment group for analysis.

Whole cell patch clamp recordings. All experiments were conducted at room temperature (22 – 24 ^{0}C) using an EPC-10 patch clamp amplifier (HEKA Electronik GmbH, Lambrecht, Germany) and using the Pulse V 8.8 acquisition program (HEKA Electronik GmbH, Lambrecht, Germany). Currents were elicited by -110 mV test pulse. The pipette solution contained (in mM): 135 $CsSO_3CH_3$, 8 NaCl, 2 $MgCl_2$, 0.5 $CaCl_2$, 1EGTA and 10 Hepes, pH 7.2. The bath solution contained (in mM): 145 NaCl, 2 $CaCl_2$, 1 MgC_2, 10 HEPES, pH 7.4. Whole cell current was analyzed using IGOR software (WaveMetrics, Lake Oswego, OR).

Surface protein isolation. For VE-cadherin internalization experiments, passage 5 HLMVEC were grown on 10 cm gelatin coated plates to confluency. The Pierce™ Cell Surface Protein Isolation Kit was used according to the package instructions with the following modifications. One 10 cm plate of cells was used per condition, with conditions being DMSO vehicle or 5, 15 or 30 min of 5 μM Yoda1 treatment. Following Yoda1 or vehicle treatment, cells were washed

quickly with HBSS with Ca^{2+} and Mg^{2+} before adding the EZ-Link Sulfo-NHS-SS-Biotin, also prepared in HBSS with Ca^{2+} and Mg^{2+}. Cells were lysed in 250 μL lysis buffer and sonicated briefly, 5 pulses at the lowest setting (10%) for 1 s each on ice [instrument info]. Lysates were incubated on ice for an additional 30 min before spinning down. Then, 200 μL of cell lysate was added to each column prepared with 400 μL NeutrAvidin Agarose slurry and placed in an end-over-end rotator for 1 h. The first flow through fraction was collected as the intracellular fraction, and then the column was washed 3 times. Then, 200 μL of 2x Laemmli buffer with Dithiothreitol (DTT) was then added to the columns, which were rotated for 1 h at room temperature to elute the biotinylated proteins. For western blotting, 15 μL of the biotinylated surface fraction was loaded on the gel after boiling for 5 min. For the intracellular fraction, 15 μL of lysate was mixed with 5 μL 4x Laemmli, with a total of 20 μL loaded on the gel after boiling for 5 min.

Immunofluorescence staining Human lung microvascular cells at passages 4 or 5 were seeded onto gelatin-coated glass coverslips and cultured in EGM2 for 3 days to achieve a confluent monolayer. Cells were treated for 30 min with DMSO vehicle or 3 μM Cells were washed twice in HBSS with Ca and Mg and fixed in 4% PFA in HBSS for 15 min at room temperature. Fixed cells were permeabilized with 0.1% triton-x100 for 30 min and blocked for 2 hours in 3% BSA. The fixed cells were then incubated with 5 μg/mL VE-cadherin antibody (ab33168, abcam) for 1 h at room temperature, washed for 10 min, three times in PBS and then incubated in anti-rabbit secondary antibody 1:200, and Alexa Fluor 647 Phalloidin (ThermoFisher Scientific) 1:200 for 1 h at room temperature, protected from light. Cells were then washed for 10 min, three times before mounting onto microscope slides with ProLong® Gold antifade reagent with DAPI. Slides were imaged with the Zeiss LSM 880 Confocal microscope. Images were analyzed using ImageJ by measuring the total area of VE-cadherin positive staining at cell-cell junctions and dividing by the total length of junctions per 63× field of view.

Co-Immunoprecipitation

Monolayers of HLMVEC passage 4 to 5 were treated with 5 μM Yoda1 for 0, 15 or 30 min. Monolayers were washed in HBSS with Ca^{2+} and Mg^{2+} briefly, then monolayers were lysed in 500 μL/10 cm plate of a buffer consisting of 10 mM Tris/Cl pH 7.5,150 mM NaCl, 0.5 mM Ethylenediaminetetraacetic acid (EDTA) and 0.5% nonyl phenoxypolyethoxylethanol (NP-40) with protease inhibitors. Lysates were kept on ice for 30 min, pipetting up and down every 10 min before centrifugation at 16,000 x g for 15 min. For immunoprecipitation, 300 μg of protein of each sample were added to a tube with 1 μg VE-cadherin antibody (Abcam, ab33168) in a total volume of 500 μL and rotated end-over-end overnight at 4 C. The following day, 40 μL of

protein G agarose bead slurry was added to each sample, and rotated end-over-end for 2 h before centrifugation at 2500 x g for 2 min. Supernatant was discarded and beads were washed 3 times in 500 μL wash buffer consisting of 10 mM Tris/Cl pH 7.5,150 mM NaCl and 0.5 mM EDTA with protease inhibitors. Protein was eluted from agarose beads by heating in 2x Laemmli buffer with 2-mercaptoenthanol for 10 min at 95 C. Blots were probed with anti-VE-cadherin (Santa Cruz, BV9) and anti-p120-catenin (Santa Cruz, sc1101).

Western blotting of whole lung lysates.

Mouse lungs were surgically removed and washed in cold PBS, and homogenized on ice in PBS containing protease inhibitors. Lysis buffer (50 mM HEPES [pH 7.4], 50 mM NaCl, 1% Triton X-100, 5 mM EDTA, 1 mM DTT, 1 mM PMSF, 10 μg/ml aprotinin, 10 μg/ml leupeptin, 10 mM sodium pyrophosphate, 50 mM sodium fluoride, and 1 mM sodium orthovanadate) was added to the lung homogenates for 1 hour. Samples were separated by SDS-PAGE, transferred to PVDF membranes and incubated overnight with anti-p120 (Santa Cruz, sc1101), β-catenin (Sigma, SAB4300470), and VE-cadherin (Santa Cruz, sc-6458) antibodies. After incubation with secondary antibodies, proteins were detected by enhanced chemiluminescence. Quantification of band intensities by densitometry was carried out using ImageJ software.

Calpain activity

Calpain activity was analyzed by the calpain activity assay kit (Abcam, ab65308). The fluorometric assay detects cleavage of calpain substrate Ac-LLY-AFC. In brief, mice lungs were isolated following PBS perfusion, and homogenized in the supplied extraction buffer. Protein concentration was determined by BCA methods. Subsequently 100 μg of samples were loaded to a 96 well black coated polystyrene plate. After addition of reaction buffer and substrate, the plate was incubated for 60 min at 37 ºC. After incubation the fluorescence units of each well was measured with excitation at 400 nm and emission at 505 nm. Signals were then normalized to the sample protein concentration. Data were shown as the relative calpain activity.

Statistics

Statistical analysis was completed using GraphPad Prism7. One-way ANOVA corrected for multiple comparisons with Tukey's test was used for experiments with more than 2 groups. Student's t-test was used for experiments with two experimental groups.

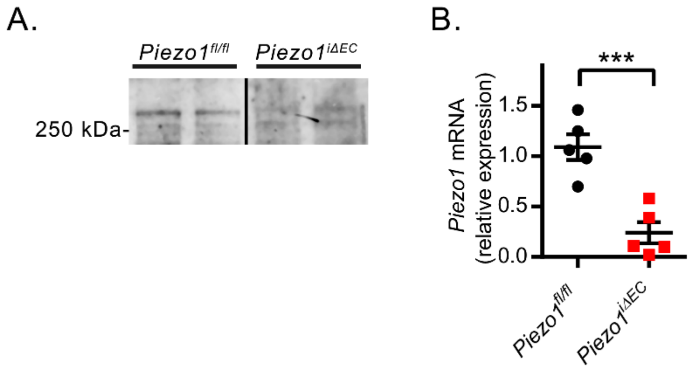

Fig. S1. Validation of endothelial specific Piezo1 deletion in mice. Mice were studied 4 weeks after the final tamoxifen injection. ECs were isolated from *Piezo1*$^{ifl/fl}$ *and Piezo1*$^{i\Delta EC}$ mice. **(A)** Western blot and RT-PCR analyses **(B)** were used to verify Piezo1 deletion in lungs. Individual data and mean ± SEM. ***, $p \leq 0.001$

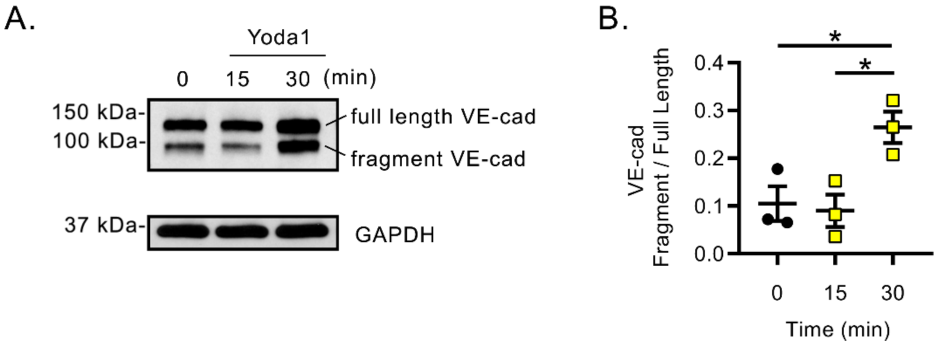

Fig. S2. Piezo1 activation with Yoda1 induces fragmentation of VE-cadherin. WB analysis (A) and quantification (B) of 90 kDa cleavage fragment of VE-cadherin in EC monolayers treated with Yoda1 for the indicated times. Individual data and mean ± SEM. *, $p \leq 0.05$.

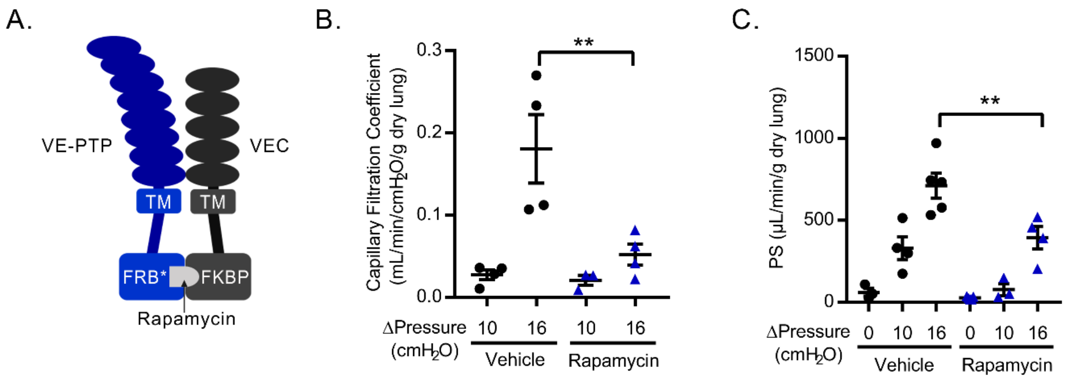

Fig. S3. Genetic stabilization of VE-cadherin junctions in mice prevents lung vascular hyper-permeability induced by increased lung microvessel pressure. **(A)** Schematic presentation of method for stabilization of AJs in mice. Knock-in mice expresses the fusion proteins VE-cadherin-FK 506 binding protein and VE-PTP-FRB* under the control of VE-cadherin (VEC) promotor. Rapamycin binds FK 506 binding protein and FRB* domains, stabilizing interaction of VE-cadherin and VE-PTP. **(B, C)** Stabilization of VE-cadherin interaction with VE-PTP significantly reduced pressure-induced increase in lung vascular permeability to albumin (B) and fluid (C). Individual data and mean ± SEM. **, p ≤0.01.

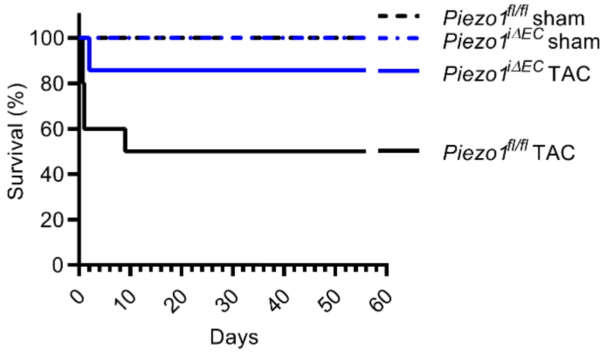

Fig. S4. Mortality in mice after transverse aortic constriction (TAC). Deletion of Piezo1 in ECs markedly increases survival in mice in response to TAC; n = 5 mice.

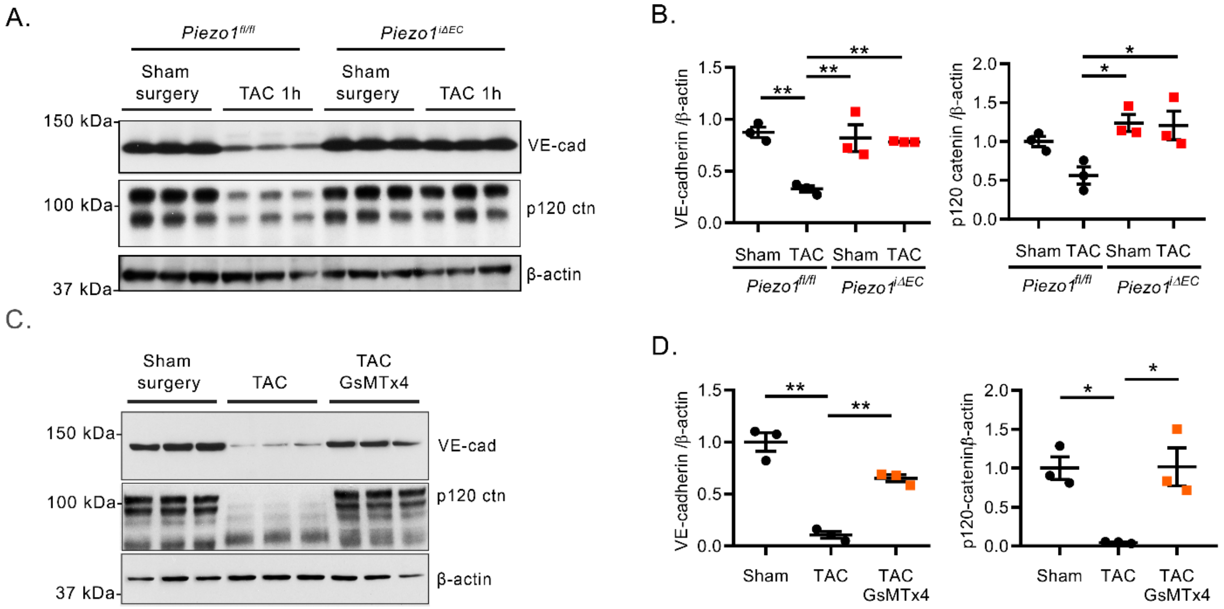

Fig. S5. Piezo1 activation in response to rise in hydrostatic pressure in mice subjected to TAC reduces expression of VE-cadherin and p120-catenin. **(A,B)** Loss of VE-cadherin and p120-catenin is mitigated in *Piezo1*$^{i\Delta EC}$ mice at 1 h post-TAC. (**C,D**) Inhibition of Piezo1 with GsMTx4 prevent loss VE-cadherin and p120 catenin in lung of mice subjected to TAC surgery for 24 h. Individual data and mean ± SEM. *, $p \leq 0.05$; **, $p \leq 0.01$

Supporting References

1. Gothert JR, *et al.* (2004) Genetically tagging endothelial cells in vivo: bone marrow-derived cells do not contribute to tumor endothelium. *Blood* 104(6):1769-1777.
2. Li J, *et al.* (2014) Piezo1 integration of vascular architecture with physiological force. *Nature* 515(7526):279-282.
3. Broermann A, *et al.* (2011) Dissociation of VE-PTP from VE-cadherin is required for leukocyte extravasation and for VEGF-induced vascular permeability in vivo. *J Exp Med* 208(12):2393-2401.
4. Cheng KT, *et al.* (2017) Caspase-11-mediated endothelial pyroptosis underlies endotoxemia-induced lung injury. *J Clin Invest* 127(11):4124-4135.
5. Vogel SM, *et al.* (2000) Abrogation of thrombin-induced increase in pulmonary microvascular permeability in PAR-1 knockout mice. *Physiol Genomics* 4(2):137-145.
6. Zaw AM, Williams CM, Law HK, & Chow BK (2017) Minimally Invasive Transverse Aortic Constriction in Mice. *J Vis Exp* (121).
7. Wang J, Ma Y, Sachs F, Li J, & Suchyna TM (2016) GsMTx4-D is a cardioprotectant against myocardial infarction during ischemia and reperfusion. *J Mol Cell Cardiol* 98:83-94.
8. Predescu DN, Neamu R, Bardita C, Wang M, & Predescu SA (2012) Impaired caveolae function and upregulation of alternative endocytic pathways induced by experimental modulation of intersectin-1s expression in mouse lung endothelium. *Biochemistry research international* 2012:672705.
